# Assessment of a Potential Role of *Dickeya dadantii* DSM 18020 as a Pectinase Producer for Utilization in Poultry Diets Based on *in silico* Analyses

**DOI:** 10.3389/fmicb.2020.00751

**Published:** 2020-04-23

**Authors:** Dana K. Dittoe, Ravi D. Barabote, Michael J. Rothrock, Steven C. Ricke

**Affiliations:** ^1^Department of Food Science and Center for Food Safety, University of Arkansas, Fayetteville, AR, United States; ^2^Department of Biological Sciences, University of Arkansas, Fayetteville, AR, United States; ^3^Egg Safety and Quality Research Unit, U.S. National Poultry Research Center, United States Department of Agriculture-Agricultural Research Service (USDA-ARS), Athens, GA, United States

**Keywords:** free range, pectinase, *Dickeya dadantii*, all-vegetable diets, *in silico*

## Abstract

Currently, the poultry industry has been faced with consumer pressure to utilize only vegetable feedstuffs in poultry diets, eliminate antibiotics from poultry production, and rear poultry in free range systems. To maintain current production standards, the industry must determine ways to enhance nutrient uptake and utilization further. One possible solution is the supplementation of pectinase, an enzyme that degrades pectin within the cell walls of plants, in poultry diets. Therefore, the objective of the current study was to determine the potential role of a pectinase producer, *Dickeya dadantii* DSM 18020, as a commercially utilized pectinase producer in poultry diets against other known pectinase producers, *in silico*. In the current study, whole genomes of *Dickeya dadantii* DSM 18020 (Dd18020), *D. dadantii* 3937 (Dd3937), *D. solani* IPO 2222 (Ds2222), *Bacillus halodurans* C-125 (BhC125), and *B. subtilis* subsp. *subtilis* str. 168 (Bs168) were compared using bioinformatic approaches to compare the chromosomal genome size, GC content, protein coding genes (CDS), total genes, average protein length (a.a.) and determine the predicted metabolic pathways, predicted pectin degrading enzymes, and pectin-degradation pathways across pectinase producers. Due to insufficient information surrounding the genome of Dd18020 (lack of annotation), the genome of Dd3937, a 99% identical genome to Dd18020, was utilized to compare pectinase-associated enzymes and pathways. The results from the current study demonstrated that Dd3937 possessed the most significant proportion of pathways presented and the highest number of pathways related to degradation, assimilation, and utilization of pectin. Also, Dd18020 exhibited a high number of pectinase-related enzymes. Both Dd3937 and Dd2222 shared the pectin degradation I pathway via the EC 3.1.1.11, EC 3.2.1.82, and EC 4.2.2.- enzymes, but did not share this pathway with either *Bacillus* species. In conclusion, Dd18020 demonstrated the genetic potential to produce multiple pectinase enzymes that could be beneficial to the degradation of pectin in poultry diets. However, for Dd18020 to become a commercially viable enzyme producer for the poultry industry, further research quantifying the pectinase production *in vitro* and determining the stability of the produced pectinases during feed manufacturing are necessary.

## Introduction

*Dickeya dadantii*, previously classified as *Erwinia chrysanthemi* by Burkholder (1957), is one the many bacteria responsible for bacterial soft rot disease (*Dickeya* spp., *Erwinia* spp., *Pectobacterium* spp., etc.) that occurs in a wide range of crops (Samson et al., 2005; Toth et al., 2011). The facultative anaerobic, Gram-negative bacilli typically grow at an optimal temperature of 39°C, but can grow between the ranges of 25–40°C (Samson et al., 2005). The typical transmission of *D. dadantii* to plants is through direct contact with contaminated soil (Grenier et al., 2006). Contamination of soil can occur through several vectors such as water (irrigation, runoff, etc.), various insects, and specific agricultural techniques such as plowing (Grenier et al., 2006). Due to the pectinolytic nature of *D. dadantii*, it causes an infection in crops that induces soft rot that can be characterized by the rapid disorganization of the parenchymatous tissues (Hugouvieux-Cotte-Pattat et al., 1996). Although the colonization of plants by soft rot *Erwinia* is primarily driven by the production of pectic enzymes, the process is multifactorial requiring cellulases, iron assimilation, an Hrp type III secretion system, exopolysaccharides, motility, and proteins involved in resistance against plant defense mechanisms (Barabote et al., 2003; Toth et al., 2003; Ravirala et al., 2007). Due to the capability of *D. dadantii* to produce pectinases, it has the potential to be advantageous to multiple industries.

Pectinases consist of a group of enzymes that hydrolyze pectin present in plant cell walls and exist in higher order plants and microorganisms (Whitaker, 1991). In plants, pectinases are found to enhance cell wall extension (Jayani et al., 2005) and promote softening of specific plant tissues during maturation and later storage (Aguilar and Huirton, 1990; Sakai, 1992). Therefore, pectinases are used to degrade plant materials in multiple food processing techniques such as reducing the time it takes to extract fruit juice from fruit puree (Junwei et al., 2000) and enhancing the clarity in wine (Kobayashi et al., 2001). Currently, pectinase is applied in several processes such as textile processing and bio-scouring of cotton fibers, the degumming and retting of plant bast fibers, wastewater treatment, coffee and tea fermentation, paper and pulp industry, purification of plant viruses, and in animal feed (Sharma et al., 2013). Across industries, pectinases are mass manufactured by utilizing pectinase producing bacteria and fungi. Of such bacteria and fungi, *Bacillus and Aspergillus* are the most characterized pectinase producers utilized across manufacturing of agricultural and food products (Garg et al., 2016).

Of the many uses of pectinases, the poultry industry would greatly benefit from the addition of pectinases in poultry diets. Recently, consumer pressure has motivated the poultry industry to introduce no antibiotic ever (NAE), all -vegetable fed broilers (Weil, 2017). In addition, there has also been an increased demand for free range poultry and forage feeding. In efforts to utilize locally available feed sources, sectors of the poultry industry have initiated forage feeding, pasture farming, or supplementary feeding regimens (Buchanan et al., 2007). With the move to all -vegetable diets and forage feeding, broiler diets may contain an increased concentration of cereals containing non-starch polysaccharides (NSPs) in their endosperm (Broz and Beardsworth, 2002). Poultry are unable to produce many of the enzymes necessary to degrade NSP’s and broiler performance is decreased due to the impairment of digestive enzymes, decreased nutrient absorption, and increase in viscous excreta (Broz and Beardsworth, 2002).

The supplementation of diets with pectin has specifically resulted in the decrease of growth rate, decrease in feed efficiency, increase in feed intake, increase in sticky droppings, and overgrowth of *Clostridium* species (Wagner and Thomas, 1977, 1978; Ricke et al., 1982). More recently, research has demonstrated that pectinases have the ability to degrade pectin more readily in poultry diets alone and in combination with other NSP-hydrolyzing enzymes. In addition, the supplementation of pectinase, cellulase, or hemicellulase alone in poultry diets has proven to be less effective than their combination on improving poultry performance (Tahir et al., 2005, 2006).

Currently, the major sources of NSP-containing plant material within poultry diets is corn and soybean meal (Pierson et al., 1980; Chesson, 2001). However, due to the increasing demand for corn and soybean meal from the swine industry and other sectors, the poultry industry has explored alternative sources of carbohydrates. Such sources are wheat, barley, and lupins (Australia) which contain high amounts of NSPs compared to those contained in corn and soybean meal, specifically pectin (Bailoni et al., 2005; Leeson, 2008; Kim et al., 2011).

Therefore, there is a need for a wide variety of enzymes, especially those that break down plant material that poultry are unable to utilize on their own due to the vast differences in plant composition. For example, of the pectins contained within the cell wall of plants vary in amount and in the physiochemical properties such as structural differences among the types of plants and fruits (Kamnev et al., 1998). Pectin structure consists of an α(1-4)-linked polygalacturonans with varying chain lengths, intermittent rhamnosyl residues, and other potential structural variations (Selvendran, 1985; Piknik, 1990; King, 1993). Further, there are currently no products on the market that solely target the complex structures of pectins, especially those contained in lupins (Kim et al., 2011). Pectin contain galactose residues of the polygalacturonans that are methyl-esterfied at C-6, which protects the substrate from the access of pectinases (Ali et al., 2005). Further, the *in vitro* supplementation of pectin methyl esterase and polygalacturonase in combination with ground dehulled lupins (*Lupinus angustifolius* in 70 mL acid buffer) reduced the length of pectin chains by 65% and molecular weight of pectin by 56% (Ali et al., 2005). As such, it is imperative to identify bacterial enzyme sources capable of producing a wide variety of pectinases for potential commercial development.

Pectinases have been provided in poultry diets in addition to other enzymes to enhance nutrient digestibility of broilers and other poultry species. Previous research has demonstrated that the use of pectinases in an enzyme cocktail has the capability to reduce the viscosity of the feed, release nutrients through the hydrolysis of non-biodegradable fibers or those blocked by these fibers and reduce the total amount of excreted feces (Jayani et al., 2005). Therefore, the overall objectives of the current study were to determine if the genome of *Dickeya dadantii* DSM 18020 (NZ_CP023467.1) encodes pectinases, if, in turn, those pectinases are comparable to those currently utilized in the poultry industry, and if *D. dadantii* would make a suitable alternative source of novel pectinases to those bacteria currently used commercially to produce pectinases.

## Materials and Methods

### Genomic Data Retrieval

In the current study, genomic data was accessed and collected from the National Center for Biotechnology Information (NCBI) on December 7, 2018 (O’Leary et al., 2016). The genomic data for *Dickeya dadantii* DSM 18020 (NZ_CP023467.1), *D. dadantii* 3937 (NC_014500.1; Glasner et al., 2011), *D. solani* IPO 2222 (NZ_CP015137.1; Pédron et al., 2014), *Bacillus subtilis* subsp. *subtilis* str. 168 (NC_000964.3; Kunst et al., 1997; Barbe et al., 2009; Belda et al., 2013; Borriss et al., 2018) and *B. halodurans* C-125 (NC_002570.2; Takami et al., 2000), was utilized in the current study to compare novel pectinase homologs (*Dickeya* spp.) to known industrialized pectinase producers (*Bacillus* spp.) and their associated metabolic pathways.

### Genomic Relation Across Pectinase Producers

Genomic data that was retrieved from NCBI was utilized to compare the genomic characteristics of *Dickeya dadantii* DSM 18020 (Dd18020), *D. dadantii* 3937 (Dd3937), *D. solani* IPO 2222 (Ds2222), *Bacillus subtilis* subsp. *subtilis* str. 168 (Bs168), and *B. halodurans* C-125 (BhC125). Genomic characteristics that were compared among bacterium were chromosomal genome size (Mb), Guanidine to Cytosine content (GC Content; %), protein-coding genes (CDS), total genes, and average protein length (a.a).

Dd3937, a wild-type strain originally isolated from *Saintpaulia ionantha*, is most commonly used as a model organism for soft rot pathogenesis since the 1980’s (Glasner et al., 2011). As Dd3937 is a known model organism, the authors wanted to explore the candidacy of a more recently discovered strain, DSM 18020. Dd18020 was recently isolated from *Pelargonium capitum* in Comoros and deposited to the Leibniz Institute DSMZ-German Collection of Microorganisms and Cell Culture (Braunschweig, Germany). The authors were particularly motivated to choose Dd18020 as the candidate pectinase producer in the current study as there are no current Nagoya Protocol restrictions to its use, it is obtainable through multiple collections of plant-associated bacteria, and has a complete genome assembly available on NCBI. Therefore, Dd18020 can be annotated (genome), purchased, and applied in research or industrial use consistently and without restrictions.

However, due to the absence of Dd18020 in several databases, Dd18020 and Dd3937 were compared against one another by comparing syntany and homogeneity. Syntany was explored using the progressive MAUVE whole genome alignment (Darling et al., 2004). Homogeneity was investigated using Web Blast on NCBI (Altschul et al., 1990). As other research has utilized reference genomes to identify close proximity to the target bacteria or organism (Tyson et al., 2004; Iverson et al., 2012; Wrighton et al., 2012; Albertsen et al., 2013; Sharon et al., 2013), the comparison of Dd18020 and Dd3937 was utilized to validate if Dd3937 was an appropriate reference genome for Dd18020. Therefore, further genomic comparisons were done in the absence of Dd18020, with Dd3937 in its stead. In addition, a rooted phylogenetic tree was constructed from the output obtained from progressiveMAUVE whole genome alignment of Dd18020, Dd3937, Ds2222, Bs168, and BhC125 (Darling et al., 2010).

### Predicted Pathways Across Pectinase Producers

In the current study, genomes of Dd3937, Ds2222, Bs168, and BhC125 were compared in MetaCyc (MetaCyc 22.5; Caspi et al., 2014) to evaluate the protein and pathway similarities between genomes of the four pectinase producers. Numerical comparisons were performed in Microsoft Excel for Office 365 MSO (16.0.11601.20174; Microsoft, Redmond, WA, United States) to highlight the numerical difference in pathways between the four pectinase producers utilized in the current study.

### Predicted Pectin-Related Enzymes Across Pectinase Producers

The list of proteins and enzymes encoded in the genomes of Dd18020, Dd3937, Ds2222, Bs168, and BhC125 was retrieved from NCBI (O’Leary et al., 2016) and utilized to determine the pectin-related proteins and enzymes encoded. Proteins encoded in the genome of the pectinase producers were aligned in MegaX (Kumar et al., 2018) using ClustalW (Thompson et al., 1994) where Maximum Likelihood was utilized to produce a rooted phylogenetic tree. In addition, Metacyc was subsequently utilized to compare pectinase related enzymes and genes across the four pectinase producers: Dd3937, Ds2222, Bs168, and BhC125. The top eleven related proteins were chosen for comparison in the current study.

### Pectinase Pathway

As Dd18020 is less studied, Dd3937 was chosen to elucidate the pectin-related pathways. MetaCyc 22.5 was utilized to determine the pathways of Dd3937. Dd3937 was subsequently compared to the other pectinase producers utilized in the current study: Ds2222, Bs168, and BhC125.

## Results

**FIGURE 1:**
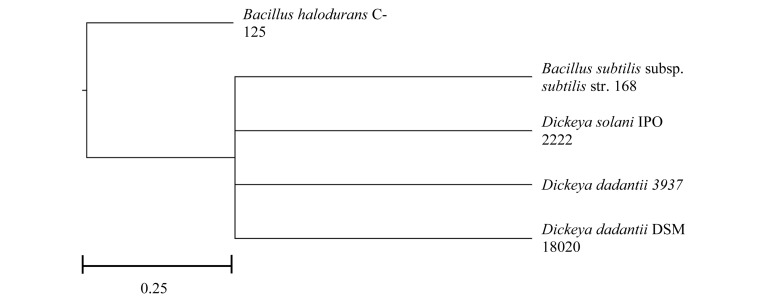
Rooted phylogenic tree of five pectinase-producing bacteria after multiple alignment in MAUVE.

In relation to the genomic make-up of the pectinase producers, all *D. dadantii* species, Dd18020 and Dd3937, and Ds2222 were more similar in their genome size (5.00, 4.92, and 4.92 Mb, respectively), GC content (56.40, 56.30, and 56.20%, respectively) and average protein-coding length (326, 323, 339 a.a., respectively) than the size, GC content, and average protein-coding length of the two *Bacillus* species (4.22 and 4.20 Mb, 43.50 and 43.70%, and 290 and 298 a.a., respectively, Table 1). All bacteria in the current study possessed similar levels of proteins and genes, with BhC125 having the fewest number of proteins and genes (3,950 CDS and 4,134 total genes). In addition, both bacilli possessed smaller average protein lengths than the *Dickeya* species. The differences in size, GC content, average protein-coding length, proteins, and genes may be attributed to *Dickeya* and *Bacillus* species belonging to different phyla (Proteobacteria and Firmicutes, respectively) (Figure 1, Galperin, 2015; Lockwood et al., 2019).

**TABLE 1:** Comparative genomic information of five pectinase producing bacteria.

**Bacterium**	**NCBI accession number**	**Chromosomal genome size (Mb)**	**GC content**	**Protein coding genes (CDS)**	**Total genes**	**Average protein length (a.a.)**	**Isolated from**	**References**
*Dickeya dadantii* DSM 18020	NZ_CP023467.1	5.00	56.40%	4,271	4,531	326	*Pelargonium capitatum*	Cheng et al., 2017
*Dickeya dadantii* 3937	NC_014500.1	4.92	56.30%	4,244	4,485	323	*Saintpaulia ionantha*	Glasner et al., 2011
*Dickeya solani* IPO 2222	NZ_CP015137.1	4.92	56.20%	4,132	4,329	339	*Solanum tuberosum*	Khayi et al., 2016
*Bacillus subtilis* subsp. *subtilis* str. 168	NC_000964.3	4.22	43.50%	4,237	4,871	290	Mutant strain of *B. subtilis Marburg^1^*	Kunst et al., 1997; Belda et al., 2013; Borriss et al., 2018; Barbe et al., 2009
*Bacillus halodurans* C-125	NC_002570.2	4.20	43.70%	3,950	4,134	298	Soil	Nakasome et al., 2000; Takami and Horikoshi, 2000; Takami et al., 2000

**FIGURE 2:**
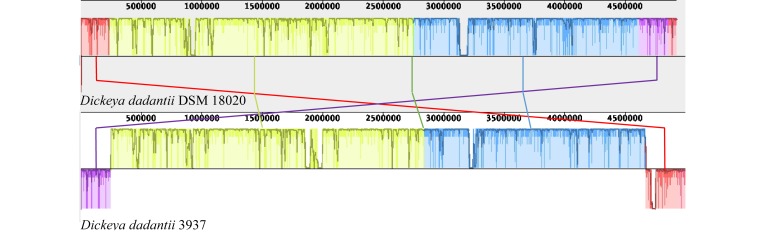
Synteny analysis of *Dickeya dadantii* DSM 18020 (NZ_CP023467.1) and 3937 (NC_014500.1). Synteny analysis was performed using MAUVE whole genome alignment. Arrows indicate the regions that are located in different positions in both genomes.

Due to the paucity of literature and available genomic research concerning Dd18020, comparison of Dd18020 and Dd3937 genomic information was performed in Web BLAST (Table 2). The results of the global alignment in Web BLAST revealed that the two genomes were a 99% match and therefore the genome of Dd3937 was sufficient to compare to other pectinase producers in lieu of Dd18020. The *E*-value, maximum score, and maximum identity of Dd18020 and Dd3937 genomes were 0.0, 1.661e + 05, and 99%, respectively, indicating that the local coverage was high. The total metrics were also relatively high, with a total score and query coverage of 9.161e + 06 and 91%, respectively. In addition, synteny was explored between Dd18020 and Dd3937 (Figure 2). The Dd18020 genome is highly syntenic with the Dd3937 genome, exhibiting only two disruptions. One region of about 250 kb is present at different locations in both chromosomes (location 4.6–4.85 Gb in Dd18020 and 0–242 kb in Dd3937). A second region in Dd18020 chromosome is split into two regions. The first region of Dd18020 is at position 0 to 228 kb and the second region at position 4.85–4.92 Gb.

**TABLE 2:** Direct comparison of *Dickeya dadantii* DSM 18020 and 3937 complete genomes.

**Description**	**Max score**	**Total score**	**Query cover**	***E-*value**	**Ident**	**Accession**
*Dickeya dadantii* 3937, complete genome	1.661e + 05	9.161e + 06	91%	0.0	99%	NC_014500.1

The metabolic pathways of the four pectinase producers were compared in MetaCyc 22.5, with Dd3937 representing Dd18020. The pathways did differentiate in several ways (Table 3). In all, Dd3937 and Bs168 exhibited the highest representation of pathways, however, Dd3937 had the highest proportion of total pathways presented. In relation to degradation, assimilation, and utilization, Dd3937 possessed the highest number of related pathways. Carbohydrate biosynthesis was present in the highest number (14) in Dd3937 when compared to the three other pectinase producers. Ds2222 had the second highest number of carbohydrate biosynthesis pathways (12) than both *Bacillus* species (8 and 6); however, when carbohydrate degradation pathways were examined, Dd3937 had the lowest representation of pathways (17), with Ds2222 and Bs168 possessing more carbohydrate degradation pathways (20 and 20, respectively).

**TABLE 3:** Analysis of metabolic pathways in the genomes of four pectinase producing bacteria.

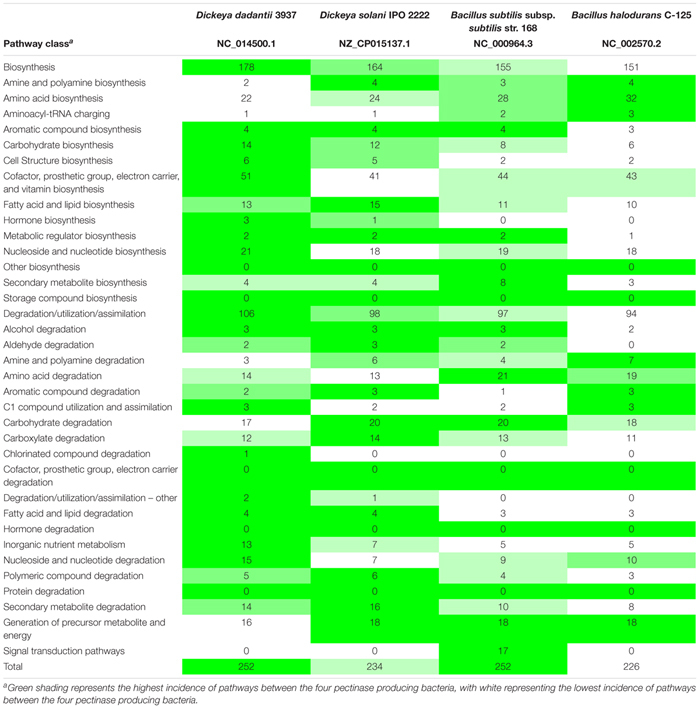

**FIGURE 3:**
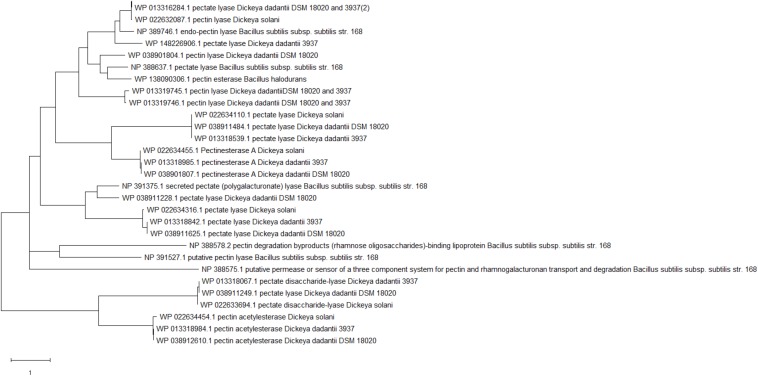
Rooted phylogenic tree of pectin degrading enzymes encoded in the genomes of *Dickeya dadantii* 18020, *D. dadantii* 3937, *D. solani* IPO 2222, *Bacillus subtilis* subsp. *subtilis* str. 168, and *B. halodurans* C-125. Proteins encoded in the genome of the pectinase producers were aligned in MegaX using ClustalW where Maximum Likelihood was utilized to produce a Newick tree.

Dd18020 was screened for pectinase-related genes by exploring the genome in NCBI (Table 4). The genome of Dd18020 contained 13 identified extracellular pectinase-related proteins: pectin acetylesterase, pectinesterase A, MULTISPECIES: pectin degradation protein KdgF, and several pectate lyases. The pectin degrading enzymes and proteins encoded in the genome of the five pectinase producers in the current study demonstrated no distinct pattern in genetic divergence (Figure 3). For example, Dd18020, Dd3937, Ds2222, and Bs168 possess the genetic potential to produce pectin lyase which evolutionarily is the most recent with Dd18020, Dd3937, and Ds2222 being a polytomy. Overall, the evolutionary divergence of pectin degrading enzymes is primarily due to the emergence of new proteins and enzymes, rather than the differences between pectinase producing bacteria. Pectin degrading enzymes encoded by *Dadantii* species were less divergent between one another than those encoded by the Bacilli.

**TABLE 4:** Pectin degradation related enzymes produced by *Dickeya dadantii* DSM 18020.

**NCBI accession number**	**Protein length**	**Protein description**
WP_038911228.1	576	Pectate lyase
WP_038912610.1	551	Pectin acetylesterase
WP_038901807.1	366	Pectinesterase A
WP_013318064.1	110	MULTISPECIES: pectin degradation protein kdgF
WP_013316284.1	315	Pectate lyase
WP_038911625.1	344	Pectate lyase
WP_013319746.1	374	Pectate lyase
WP_013319745.1	375	Pectate lyase
WP_038911695.1	392	Pectate lyase
WP_038901806.1	392	Pectate lyase
WP_038901804.1	404	Pectate lyase
WP_038911484.1	425	Pectate lyase
WP_038911249.1	543	Pectate lyase

Due to the paucity of genomic research related to Dd18020, Dd3937 was compared to the three other pectinase-producing bacteria in MetaCyc 22.5 (Table 5). The top eleven pectinase-related enzymes that were shared among pectinase producers are also present in Dd18020 (Tables 4, 5), however, Ds2222 did not appear to share similar enzymes to either Dd3937 or both *Bacillus* species. It appears that *D. dadantii* possesses more proteins dedicated to degradation, utilization, and assimilation (105) than Ds2222, Bs168, and BhC125 (97, 95, and 94 proteins, respectively). However, *in vitro* modeling should be done to quantify the pectinase production of *D. dadantii* compared to other pectinase-producing bacteria.

**TABLE 5:** Comparison of key pectinase enzymes between four pectinase producing bacteria.

**Protein homolog**		***Dickeya dadantii* 3937**	***Dickeya solani* IPO 2222**	***Bacillus subtilis* subsp. *subtilis* str. 168**	***Bacillus halodurans* C-125**
1	Product	DDA3937_RS15625	None	YesY	AYT26_RS19885
	Gene	Pectin acetylesterase	None	Rhamnogalacturonan acetylesterase	Hypothetical protein
2	Product	DDA3937_RS11325	None	None	AYT26_RS02680
	Gene	Pectin degradation protein kdgF	None	None	Cupin
3	Product	DDA3937_RS15615	None	Pel	AYT26_RS03755
	Gene	Pectate lyase	None	Pectate lyase	Hypothetical protein
4	Product	DDA3937_RS16955	None	yesO	AYT26_RS05885
	Gene	Sugar ABC transporter substrate-binding protein	None	Pectin degradation byproducts-binding lipoprotein	Hypothetical protein
5	Product	DDA3937_RS19500	None	None	AYT26_RS19165
	Gene	Pectate lyase	None	None	pectate lyase
6	Product	DDA3937_RS11370	None	ytmA	AYT26_RS16630
	Gene	Pectin acetylesterase	None	Putative hydrolase	Peptidase
7	Product	DDA3937_RS02860	None	pelB	None
	Gene	Pectate lyase	None	Pectin lyase	None
8	Product	DDA3937_RS11370	None	ytmA	AYT26_RS16630
	Gene	Pectin acetylesterase	None	Putative hydrolase	Peptidase
9	Product	DDA3937_RS09860	None	ywoF	None
	Gene	Pectate lyase	None	Putative pectate lyase	None
10	Product	DDA3937_RS11105	None	pelC	None
	Gene	Type III secreted protein	None	Secreted pectate lyase	None
11	Product	DDA3937_RS15625	None	yesY	AYT26_RS19885
	Gene	Pectin acetylesterase	None	Rhamnogalacturonan acetylesterase	Hypothetical protein

**FIGURE 4:**
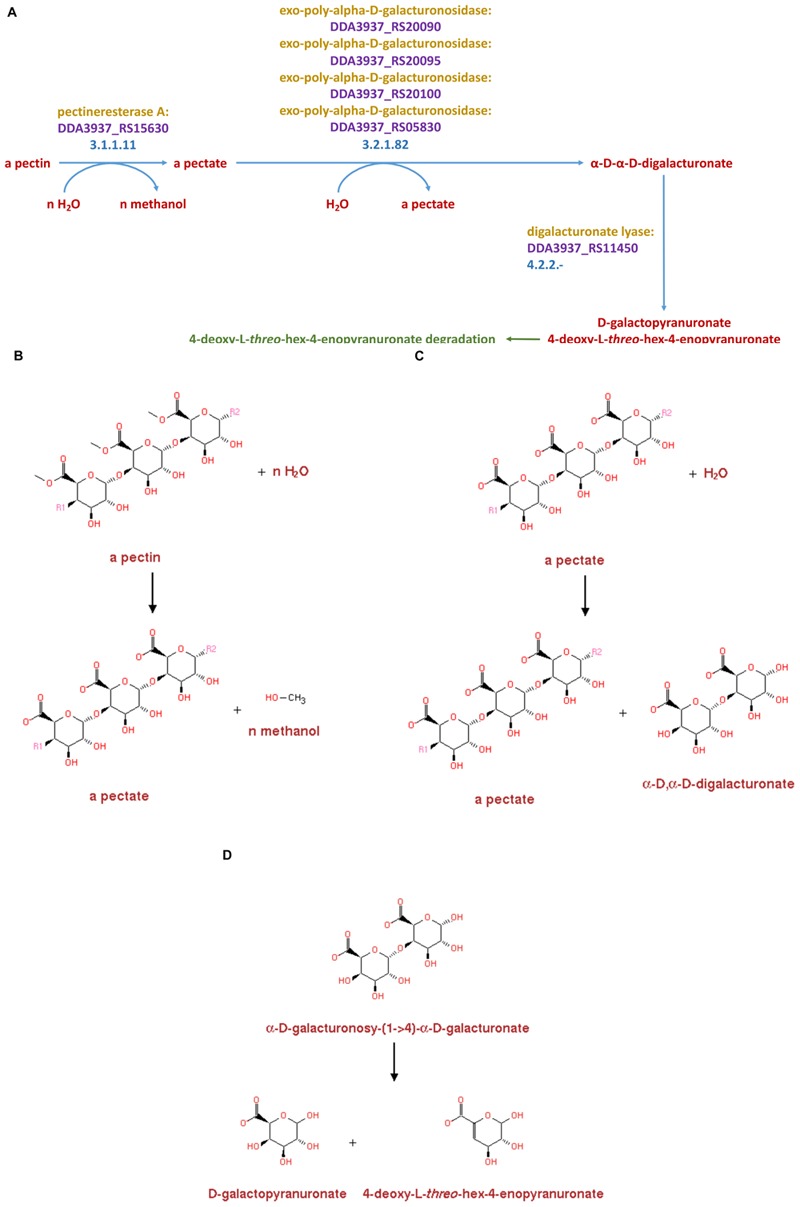
Enzymes, genes, and products for pectin degradation in *Dickeya dadantii* 3937 **(A)**. Pectin is first acted upon by pectinesterase A (3.1.1.11) which yields a pectate and n-methanol **(B)**. Pectate is further degraded to α-D-α-D-digalacturonate by an exo-poly-α-D-galacturonosidase (3.2.1.82) with a pectate as a byproduct **(C)**. The α-D-α-D-digalacturonate is then degraded to D-galactopyranuronate 4-deoxy-L-threo-hex-4-enopyranuronate by the enzyme catalyst digalacturonate lyase (4.2.2.-) **(D)**

In addition to comparing the pectinase-related proteins produced by Dd18020 and the four other bacteria utilized in the current study, the metabolic pathways related to pectin degradation were compared simultaneously for shared pectinase related pathways in MetaCyc 22.5. Of the shared pathways, only the two *Dickeya* species shared similar pathways, EC 3.1.1.11, EC 3.2.1.82, and EC 4.2.2.- (Figure 4A). The presence of shared pathways between the two *Dickeya* species was relatively surprising, as the genome-encoded pectinase-related enzyme comparison did not reveal similarities in the proteins encoded by the two *Dickeya* species. In fact, Ds2222 did not possess similarly coded proteins to that of the other three bacteria (Table 5). However, the metabolic pathway related to pectin degradation I was shared between Dd3937and Ds2222.

Pectinases have been divided into three separate groups dependent on the cleavage site. These three groups of pectinases are comprised of (1) hydrolases consisting of polygalacturonase, PG (EC 3.2.1.15); (2) lyase/trans-eliminases comprising pectin lyase, PNL (EC 4.2.2.10), and pectate lyase, PL (EC 4.2.2.2); and (3) pectinesterase, PE (EC 3.1.1.11) (Yadav et al., 2009; Visser et al., 2004). In the current study, similar pathways (Figures 4A–D) of all categories were observed in Dd18020, Dd3937, and Ds2222.

In the pectin degradation pathway, both Dd3937and Ds2222 possessed the same enzyme, pectinesterase A (EC 3.1.1.11), encoded for by the DDA3937_RS15630 and DSOIPO2222_RS15080 genes (data not shown). In the same pathway, the enzymes and genes of similar relation from Dd3937and Ds2222 were several exo-poly-α-D-galacturonosidases (EC 3.2.1.82) encoded for by the DDA3937_RS20090, DDA3937_RS20095, DDA3937_RS20100, and DDA3937_RS05830 genes of Dd3937and the DSOIPO2222_RS19410, DSOIPO2222_RS19420, and DSOIPO2222_RS19425 genes of Ds2222. Lastly, in the metabolic pathway EC 4.2.2.-, the enzymes, and genes of similar relation from Dd3937and Ds2222 were oligogalacturonate lyase encoded in the genes DDA3937_RS11450 and DSOIPO2222_RS11150, respectively. Neither *Bacillus* species utilized in the current study shared any similar enzymes or genes in the pectin degradation I pathway to the *Dickeya* species.

The pectin degradation pathway I shared by the *Dickeya* species degrades pectin to D galactopyranuronate + 4-deoxy-L-threo-hex-4-enopyranuronate (Figure 4A). Pectin is degraded to pectate via pectinesterase A in the metabolic reaction catalyzed by EC 3.1.1.11, with methanol byproduct (Figure 4B). Pectate is degraded to α-D, α-D-digalacturonate via several exo-poly-α-D-galacturonosidases in the metabolic reaction catalyzed by EC 3.2.1.82 with a pectate byproduct (Figure 4C). The α-D, α-D-digalacturonate is degraded to D galactopyranuronate + 4-deoxy-L-threo-hex-4-enopyranuronate in the metabolic pathway catalyzed by EC 4.2.2.- in Dd3937 via oligogalacturonate lyase (Figure 4D).

## Discussion

As pectinase is a vital enzyme utilized in the degradation of different fruit and vegetable products, it has value to the food industry (Sharma et al., 2013). Likewise, based on consumer preference, poultry industry diets are increasingly becoming vegetable-based void of animal protein (Weil, 2017). To make all-vegetable diets commercially viable, the poultry industry will need additional sources of feed grade pectinases with different hydrolytic capabilities to break down vegetable products with widely different compositional levels and subsequently improve broiler performance. Therefore, the utilization of a more flexible set of supplementary enzymes will become increasingly crucial to poultry nutritionists in enhancing the nutrient uptake in all-vegetable diets.

However, as *D. dadantii* has been reported to be a pathogen to plants and insects, another potential strategy for use of *D. dadantii* DSM 18020 pectinase genes would be to incorporate them into a current commercial poultry probiotic. Therefore, the genes of pectin degrading enzymes of Dd18020 could be inserted into a *Bacillus*, a known probiotic currently utilized in poultry diets. A *Bacillus* spp. would be a suitable candidate as they have the potential to withstand feed manufacturing conditions due to spore forming capabilities and can be genetically modified for industrial use (Ricke and Saengkerdsub, 2015). Though it is feasible, the insertion of Gram-negative genes (*D. dadantii*) into a Gram-positive organisms (*Bacillus*) is not easily accomplished as it requires proper plasmid origin recognition, promoter recognition, and codon usage between the organisms. Another limitation to consider is that certain *Bacillus* species such as *B. subtilis* produce proteases that have been known to hinder the production of heterologous proteins (Puohiniemi et al., 1992). As such, the meticulous insertion of Gram-negative genes into *Bacillus* spp. has been possible through plasmid insertion (Luchansky et al., 1988; Ohta et al., 2005) and more recently through CRISPR-Cas9 systems (Toymentseva and Altenbuchner, 2019).

In the current study, data demonstrated that *D. dadantii* DSM 18020 possessed several pectinase-related enzymes such as polygalacturonases (PG), pectin lyase (PL), polygalacturonate lyase (PGL), and pectinesterase or pectin methylesterase (PE) (Sharma et al., 2013). The optimal pH for most endo PG, PL, PGL, and PEs are 2.5–6.0, 4.0–7.0, 6.0–11.0, and 4.0–7.0, respectively (Sharma et al., 2013). The optimal temperatures range from 30 to 50°C (PGs) and 40–60°C (PEs) (Sharma et al., 2013). The internal body temperature of poultry is 41.5°C (Dawson and Whittow, 2000) and the pH of the avian gastrointestinal tract (GIT) varies from highly acidic (below 4) in the proventriculus and gizzard and neutral in the lower intestines (above 7; Sturkie, 1999). Therefore, these enzymes should function as the avian GIT is within the optimal parameters for enzyme utilization.

In fact, the *in vitro* evaluation of PG and pectin methylesterase (PME) demonstrated an improvement in the digestion of pectin and a reduction in the water holding capacity of lupins in comparison to PG or PME alone (Ali et al., 2005). Therefore, Ali et al. (2005) concluded that the inclusion of legumes such as lupin could be increased by utilizing the combination of the two pectinases in poultry diets. Furthermore, the supplementation of pectinase with either cellulase or hemicellulase in corn-soybean meal broiler diets from days 15 to 27 has demonstrated the improvement in the ileal digestibility of crude protein (CP) and organic matter and increased apparent metabolizable energy content of the diet (Tahir et al., 2006). Other researchers have also found the supplementation of a multi-enzyme preparation with pectinase improved CP digestibility (Marsman et al., 1997; Kocher et al., 2002; Saleh et al., 2005). The improvement in CP digestibility may in part be due to the degradation and subsequent digestion of pectic polysaccharides (Slominski and Campbell, 1990; Simbaya et al., 1996). Due to 10% of the protein being entrapped in the cell wall matrix of soybeans, the degradation of the cell wall via the utilization of pectinase-related enzymes may release the contained proteins (Chesson, 2001). Also, half of the NSP’s contained within the cell matrix of the soybean meal are pectic polysaccharides (Chesson, 2001). Their subsequent depolymerization may improve CP digestibility, which has been noted previously (Tahir et al., 2006).

## Conclusion

The current study validates the genetic potential of *D. dadantii* DSM 18020 as an enzymatic pectinase producer to utilize commercially within the poultry feed industry. The results demonstrated that several pectin-related enzymes are encoded in the genome of *D. dadantii*, and *D. dadantii* possesses the most significant number of pectin degradation-related pathways. Also, the study demonstrates the genomic capability for metabolic breakdown of pectin via the pectin degradation pathway belonging to *D. dadantii*; however, further studies are necessary to evaluate the capabilities of *D. dadantii* DSM 18020 as a source of commercially viable pectinase for large scale production. For example, more in-depth knowledge of pectin degradation by *D. dadantii* is necessary to quantify the pectinase production of *D. dadantii* to design scale up production. Based on the current *in silico* research study, *D. dadantii* does appear to produce pectinases that can further enhance the utilization of poultry diets and the continual supplementation via production by poultry probiotics fed to poultry may enhance nutrient absorption and subsequent performance. In relation to poultry, further analyses are necessary to determine if the supplementation of *D. dadantii* pectin-related enzymes in poultry diets is feasible. Some caution should be exercised as diet manufacturing involves steam and high temperature; this could potentially denature these key enzymes, thus reducing the effectiveness of pectin-related enzymes. Therefore, further research evaluating the survival of pectinases in poultry diets is necessary.

## Data Availability Statement

The datasets generated for this study can be found in the NCBI, Dickeya dadantii DSM 18020 (NZ_CP023467.1), *D. dadantii* 3937 (NC_014500.1), *D. solani* IPO 2222 (NZ_CP015137.1), *Bacillus subtilis* subsp. *subtilis* str. 168 (NC_000964.3) and *B. halodurans* C-125 (NC_002570.2).

## Author Contributions

DD wrote the manuscript with the assistance from SR, RB, and MR. All authors significantly contributed to the work of the current manuscript.

## Conflict of Interest

The authors declare that the research was conducted in the absence of any commercial or financial relationships that could be construed as a potential conflict of interest.
